# A synthetic biology approach for consistent production of plant‐made recombinant polyclonal antibodies against snake venom toxins

**DOI:** 10.1111/pbi.12823

**Published:** 2017-09-22

**Authors:** Jose Manuel Julve Parreño, Estefanía Huet, Asun Fernández‐del‐Carmen, Alvaro Segura, Micol Venturi, Antoni Gandía, Wei‐song Pan, Irene Albaladejo, Javier Forment, Davinia Pla, Andrés Wigdorovitz, Juan J. Calvete, Carlos Gutiérrez, José María Gutiérrez, Antonio Granell, Diego Orzáez

**Affiliations:** ^1^ Instituto de Biología Molecular y Celular de Plantas (IBMCP) Agencia Estatal Consejo Superior de Investigaciones Científicas Universidad Politécnica de Valencia Valencia Spain; ^2^ Instituto Clodomiro Picado Facultad de Microbiología Universidad de Costa Rica San José Costa Rica; ^3^ Instituto de Biomedicina de Valencia Agencia Estatal Consejo Superior de Investigaciones Científicas Valencia Spain; ^4^ Instituto de Virología CICVyA INTA Hurlingham Buenos Aires Argentina; ^5^ Research Institute of Biomedical and Health Sciences University of Las Palmas de Gran Canaria Arucas Las Palmas Canary Islands Spain

**Keywords:** recombinant polyclonal antibodies, molecular pharming, snake antivenoms

## Abstract

Antivenoms developed from the plasma of hyperimmunized animals are the only effective treatment available against snakebite envenomation but shortage of supply contributes to the high morbidity and mortality toll of this tropical disease. We describe a synthetic biology approach to affordable and cost‐effective antivenom production based on plant‐made recombinant polyclonal antibodies (termed pluribodies). The strategy takes advantage of virus superinfection exclusion to induce the formation of somatic expression mosaics in agroinfiltrated plants, which enables the expression of complex antibody repertoires in a highly reproducible manner. Pluribodies developed using toxin‐binding genetic information captured from peripheral blood lymphocytes of hyperimmunized camels recapitulated the overall binding activity of the immune response. Furthermore, an improved plant‐made antivenom (plantivenom) was formulated using an *in vitro* selected pluribody against *Bothrops asper* snake venom toxins and has been shown to neutralize a wide range of toxin activities and provide protection against lethal venom doses in mice.

## Introduction

Passive immunization (PI) with polyclonal antibodies (pAbs) purified from animal serum was first adopted to treat diphtheria in the 19th century (Graham and Ambrosino, 2015). Antibiotics and monoclonal antibodies have progressively substituted antisera‐based therapies. However, pAbs‐based antivenoms remain the only available treatment against complex toxin mixtures such as those present in snake venoms. The global venom burden has been estimated at 2.5 million envenomings and 125 000 deaths per year (Chippaux, 1998). Despite steady improvements (Gutierrez, 2014b; Gutierrez *et al*., 2011), antivenom‐based snakebite treatments are not free of side effects as a consequence of the exposure to heterologous antibody constant regions. Furthermore, antivenom supply is at constant risk due to high manufacturing costs and the low income level of target populations (Arnold, 2016; Harrison *et al*., 2009). Antivenoms made of recombinant human or humanized antibody cocktails would be a highly desirable alternative as they would reduce the adverse effects while facilitating product standardization and reproducibility. Unfortunately, manufacturing costs for complex antibody cocktails are still high because to ensure consistency they often require parallel production lines for each component of the cocktail. Conversely, simplifying the cocktail composition may compromise its efficacy due to the high diversity of toxins comprising venoms. Furthermore, it is difficult to reconcile moves towards cocktail simplification with a major trend in antivenom formulation, namely to widen the spectrum of snake venoms covered by a single treatment (Lavonas, 2012; Stock *et al*., 2007).

To overcome these limitations, we have developed a synthetic biology strategy for antivenom production based on the expression of recombinant pAbs in plants. Plant biofactories provide affordable alternative platforms for recombinant antibody production (usually known as ‘plantibodies’) (Stoger *et al*., 2002) due to their scalability, high production yields, the low risk of contamination with adventitious pathogens and lower requirements for production facility building (Paul and Ma, 2011; Paul *et al*., 2015). Our proposed strategy uses the plant chassis as a production platform and takes advantage of an intrinsic property of many plant viruses known as superinfection exclusion (SE) to ensure reproducibility. SE prevents the superinfection of cells by a second virus when they are already infected with a closely related resident virus (Julve *et al*., 2013; Soller and Epstein, 1965; Syller, 2012). An important consequence of SE is the fact that population variants in plant virus infections are not uniformly distributed but structured in a mosaic‐like pattern. Most importantly, this distribution ensures the maintenance and propagation of population diversity as compared to the fitness‐driven quasispecies‐like distributions typical of unstructured population dynamics (Elena *et al*., 2011). We have investigated the use of virus‐based expression systems for the induction of somatic expression mosaics in plant leaves, where each ‘tile’ in the mosaic functions as an independent monoclonal micro‐production line, together leading to the production of recombinant polyclonal cocktails. In this study, we show how this strategy ensures the simultaneous production of high yields of recombinant pAbs comprising hundreds of idiotypes with outstanding batch‐to‐batch reproducibility, independently of the complexity of the polyclonal composition. Most importantly, we show that this novel strategy enables the expression of a selected portion of a mammalian immunized antibody repertoire in a transiently multitransgenic plant in a highly reproducible manner. We also show that this strategy can be used to synthetize a complex plant‐made antibody cocktail able to neutralize a wide range of toxins in the *Bothrops asper* venom – medically the most important snake in Central America (Gutierrez, 2014a) – thus opening the door to the formulation of efficient and affordable plant‐made recombinant antivenoms.

## Results

### Characterization of somatic expression mosaics in *Nicotiana benthamiana*

**Figure 1:**
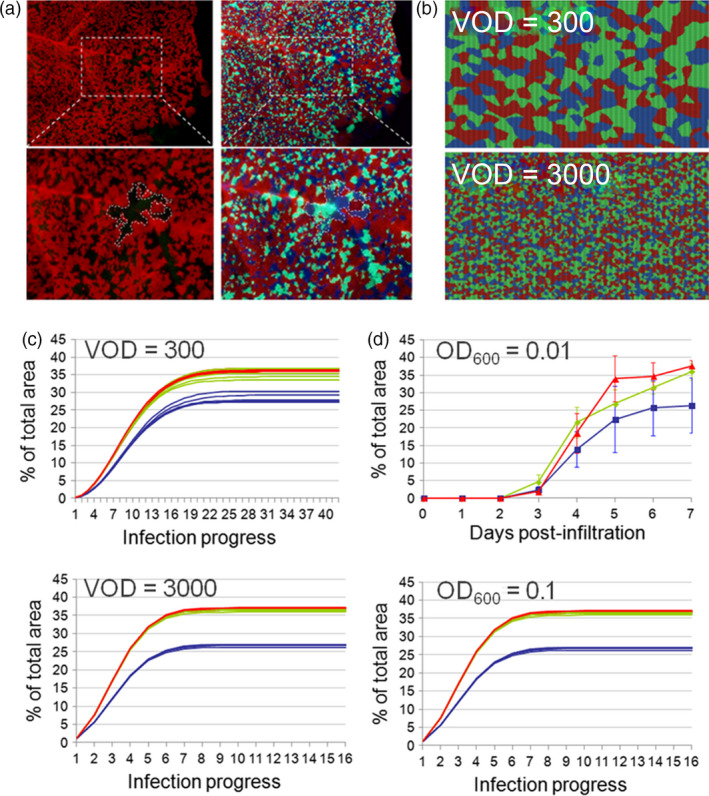
Principles of superinfection exclusion. (a) *N. benthamiana* leaves showing somatic expression mosaics produced by three magnICON viral clones encoding DsRed, green and blue florescent proteins agroinfected simultaneously at OD_600_ = 0.01. Mosaics are observed either with red/green filters for DsRed (left) or with UV light (right). Below, detail of the mosaic in the framed leaf area. (b) NetLogo computer simulation of a triple viral co‐infection (red, green and blue) subjected to SE in a virtual area comprising 62 500 virtual cells. The virtual fitness (VF) for each clone was adjusted to 1.0 : 1.0 : 0.7 (R:G:B), and virtual OD_600_ (VOD) was adjusted to 300 (upper image) and 3000 (lower image) arbitrary units to match the natural infection on (a, d) (see Methods for detailed definitions of VF and VOD). (c) Repeated NetLogo simulations of the evolution of triple R‐G‐B infections using VOD values of 300 (up) and 3000 (down), respectively. (d) Experimental data evolution of triple R‐G‐B infections in *N. benthamiana* leaves were performed at two different OD_600_ and recorded daily up to day 7 postinfiltration. Evolution of clone expansion is represented as percentage of total leaf area occupied by each clone ± SD.

The spatio‐temporal dynamics of the mosaic‐like expression patterns were monitored in *Nicotiana benthamiana* leaves after agroinfiltration of three virus‐based infective clones encoding GFP, BFP and DsRed fluorescent proteins (Figure 1a). To better understand the dynamics of mosaic formation, we created a 2D computer simulation in NetLogo (see Figure 1b and supporting information) and compared the predicted computer outputs with the experimental mosaic patterns obtained by the simultaneous agroinfiltration of three viral clones encoding fluorescent proteins. Based on the computer simulations, we anticipated that, for a given set of initial parameters, mosaic (colour) composition would be a highly reproducible outcome (Figure 1c) and that reproducibility would increase with the number of initial infection foci. Time‐course co‐infiltration analysis experimentally corroborated this prediction (Figure 1d). From these observations, we hypothesized that the (mosaic) distribution pattern of viral clones imposed by SE offers a competition‐limited microenvironment that ensures the survival of low‐fitness clones. Moreover, with a sufficiently high initial number of infective foci, SE will also protect the systems from fluctuations in population dynamics, providing highly reproducible outcomes.

### Testing expression mosaics for the production of recombinant pAbs: Pluribody technology

**Figure 2:**
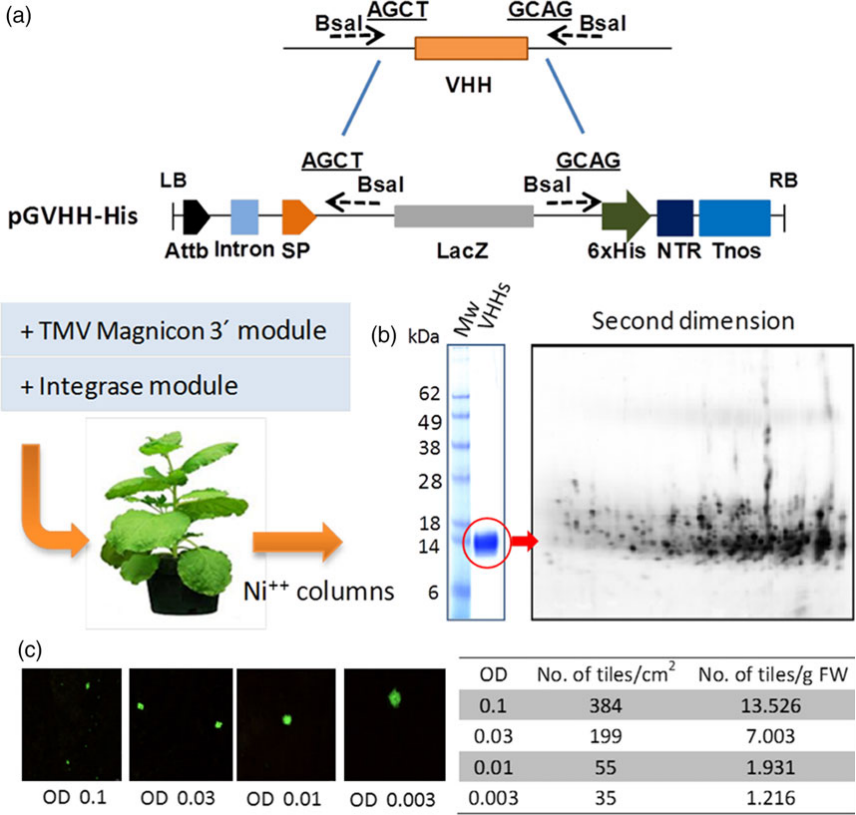
Assessment of antiserum diversity. (a) Schema of the cloning and transient expression procedures used for the production of polyclonal V_H_H antibodies. (b) First and second dimension electrophoresis separation of V_H_H‐His clones purified from *N. benthamiana* leaves using Ni‐NTA affinity columns. (c) Expression of a GFP clone as a viral subpopulation within a V_H_H ‐His library. Images were taken after 10 dpi, and the average tile size was recorded and used to estimate tile density and its dependence with OD600 (right). AttB, site‐specific recombination site; SP, signal peptide; NTR, nontranslated region; Tnos, terminator of nopaline synthase.

In a case study of a multiprotein plant biofactory, we attempted to determine whether this platform could produce high yields of recombinant antibody mixes while preserving the diversity of the composition, ensuring high batch‐to‐batch reproducibility and yielding a final product that was functionally equivalent to traditional animal antivenom. To assess diversity preservation, we first assayed the *in planta* expression of an antibody library obtained from camel peripheral mononuclear blood cells (PMBC), comprising a fraction of the antibody repertoire present in the circulating B cells of an individual dromedary camel. Camel antibodies were preferred because camelids display large immune repertoires in single chain antibody format (Hcab), a feature that greatly simplifies library construction and subsequent production in plants using viral vectors. Hcab variable antibody regions were PCR‐amplified from PMBC cDNA and cloned *en masse* into the pGV_H_H‐His vector – a modified version of tobacco‐mosaic virus (TMV)‐derived binary magnICON vector containing an in‐frame histidine tag for detection (Figure 2a) . The pGV_H_H‐His library was then mobilized to *Agrobacterium tumefaciens* cells and from here to *N. benthamiana* leaves using vacuum agroinfiltration. After 7 days’ incubation, leaf apoplast fluid was recovered from detached leaves and the V_H_H‐His were purified by affinity chromatography, resulting in a purified protein band of the expected average size (15 kDa, Figure 2b). As anticipated, a two‐dimensional electrophoretic separation resolved the underlying protein complexity and revealed a pattern of >200 distinguishable spots, indicative of the polyclonal nature of the sample (Figure 2c). The resulting plant‐made multiple recombinant antibody products are hereafter referred to as pluribody samples. To estimate the size of the repertoires that can be expressed using this system, a GFP clone was co‐infiltrated together with the pGV_H_H‐His library at different ratios. It was calculated that one gram of fresh *N. benthamiana* leaves can produce up to 13 500 expression tiles using an *Agrobacterium* library inoculum at OD_600_ = 0.1 (Figure 2c).

### Pluribody technology ensures batch‐to‐batch reproducibility

**Figure 3:**
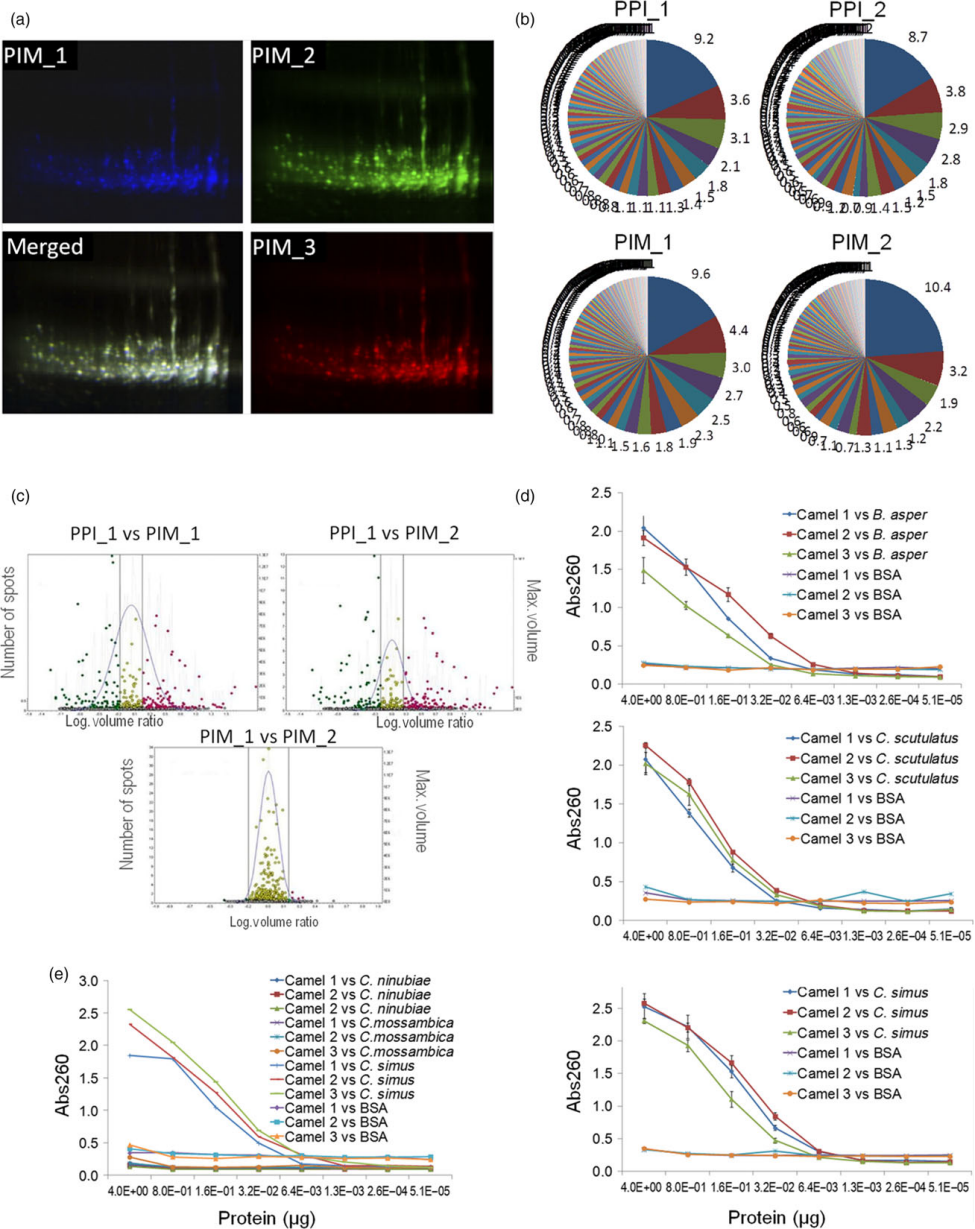
Assessment of pluribody reproducibility. (a) Individual and merged 2D DIGE images of three independent hyperimmune pluribody preparations in V_H_H‐His format (PIM_1, PIM_2 and PIM_3). (b) Comparison of the relative abundance of individual antibody clones in plant samples agroinfiltrated with inocula from independent WCBs derived from the same MCB. Antibody clones were identified by deep sequencing of V_H_H CDR3 region. PPI_1 and PP_2 correspond to equivalent pre‐immune WCBs. PIM_1 and PIM_2 correspond to equivalent immune polyclonal WCBs. In each comparison, the same colours correspond to identical antibody clones. (c). DIGE comparison of PPI and PIM purified pluribody preparations. Centred dots represent volume ratios close to one. (d) ELISA test showing the venom‐binding activity of three nonequivalent PIM preparations each obtained from a different individual camel and analysed against each of the three venoms employed in the immunization. (e) Specificity test of PIM venom binding, comparing reactivity against *Crotalus simus* and cobra venoms.

We then investigated the ability of the system to reproduce the composition and reactivity of mammalian immune subrepertoires. To this end, new antibody libraries in pGV_H_H‐His were constructed starting from PBMC samples obtained from three dromedary camels immunized with the same venom cocktail derived from three snake species, *Crotalus simus*,* Crotalus scutulatus* and *Bothrops asper*. To favour reproducibility and ensure the preservation of the original diversity in any subsequent analysis, the library construction procedure was standardized as shown in Fig. S1 (detailed in Supplementary methods). Working cell bank (WCB) aliquots were first used to evaluate the production strategy in terms of reproducibility, by comparing different V_H_H‐His pluribody preparations. First, three pluribody samples derived from the same immune repertoire but prepared from different WCBs (PIM_1, PIM_2 and PIM_3) were labelled with different fluorophores and resolved by 2D‐DIGE analysis. The almost perfect overlapping of DIGE images indicated that all three complex protein samples were remarkably similar (Figure 3a). In a separate DIGE experiment, two immune pluribodies (PIM_1 and PIM_2) were compared with a third preparation derived from a nonimmune repertoire (PPI_1). As the corresponding similarity plots show (Figure 3b), the comparison of the protein spots from equivalent immune pluribody samples (PIM_1 vs PIM_2) produced a narrowly centred volume ratio distribution indicative of a highly reproducible composition. In contrast, comparisons between samples derived from different immune repertoires (PPI_1 vs PIM_1; PPI_1 vs PIM_2) showed clear deviations in volume ratios. Furthermore, deep sequencing of the CDR3 regions in V_H_H mRNAs from agroinfiltrated plants showed the remarkable conservation of sequence distribution between equivalent samples (PPI1 vs PPI2 and PIM1 vs PIM2), with only very minor differences observed in the most abundant CDR3 sequences (Figure 3c). Finally, when the venom‐binding activities of PPI and PIM samples were compared, the immune pluribodies consistently showed reactivity against all three venoms employed for immunizations (Figure 3d), and no binding activity was observed against BSA or nonrelated *Naja* venoms (*Naja nubiae* and *Naja mossambica*) (Figure 3e).

### Formulation of a snake plantivenom using Pluribody technology

**Figure 4:**
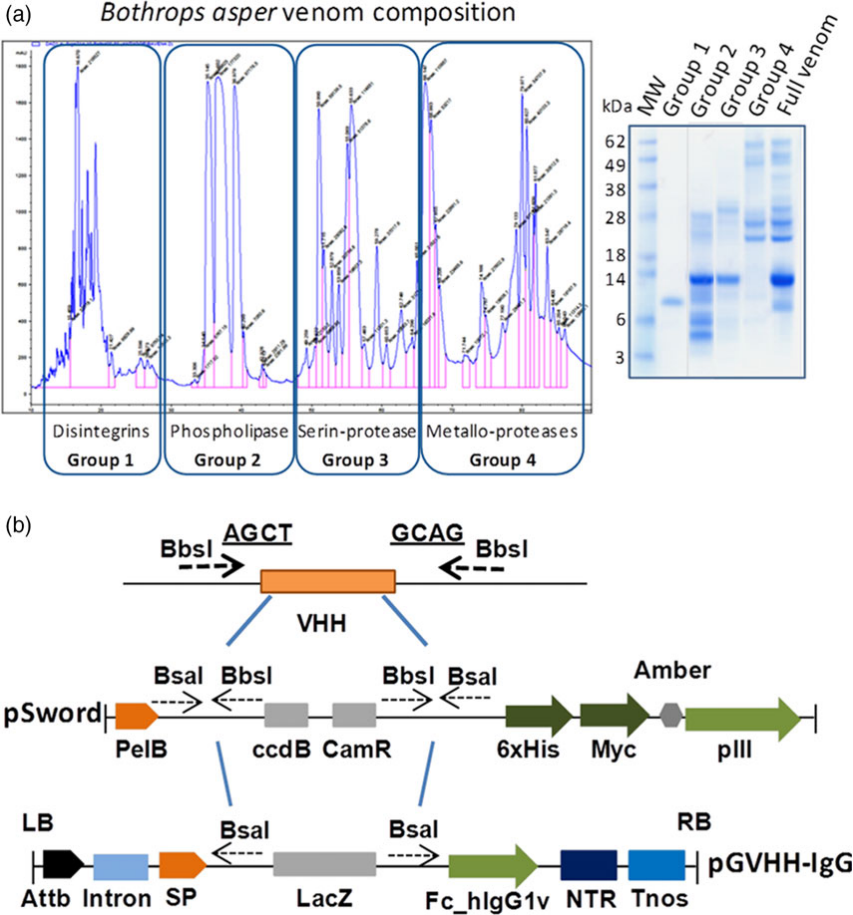
Strategy for pluribody formulation enrichment. (a) Chromatographic profile and electrophoresis separation of *Bothrops asper* venom employed in antivenom pluribody enrichment, showing the four groups of toxins employed in phage display selection to reduce antigen drift. (b) Cloning procedure for V_H_H variable regions from PBMC cDNA to pHEN2‐derived pSword phage display vector and from here to binary vector for plant expression adapted from magnICON deconstructed system.

**Figure 5:**
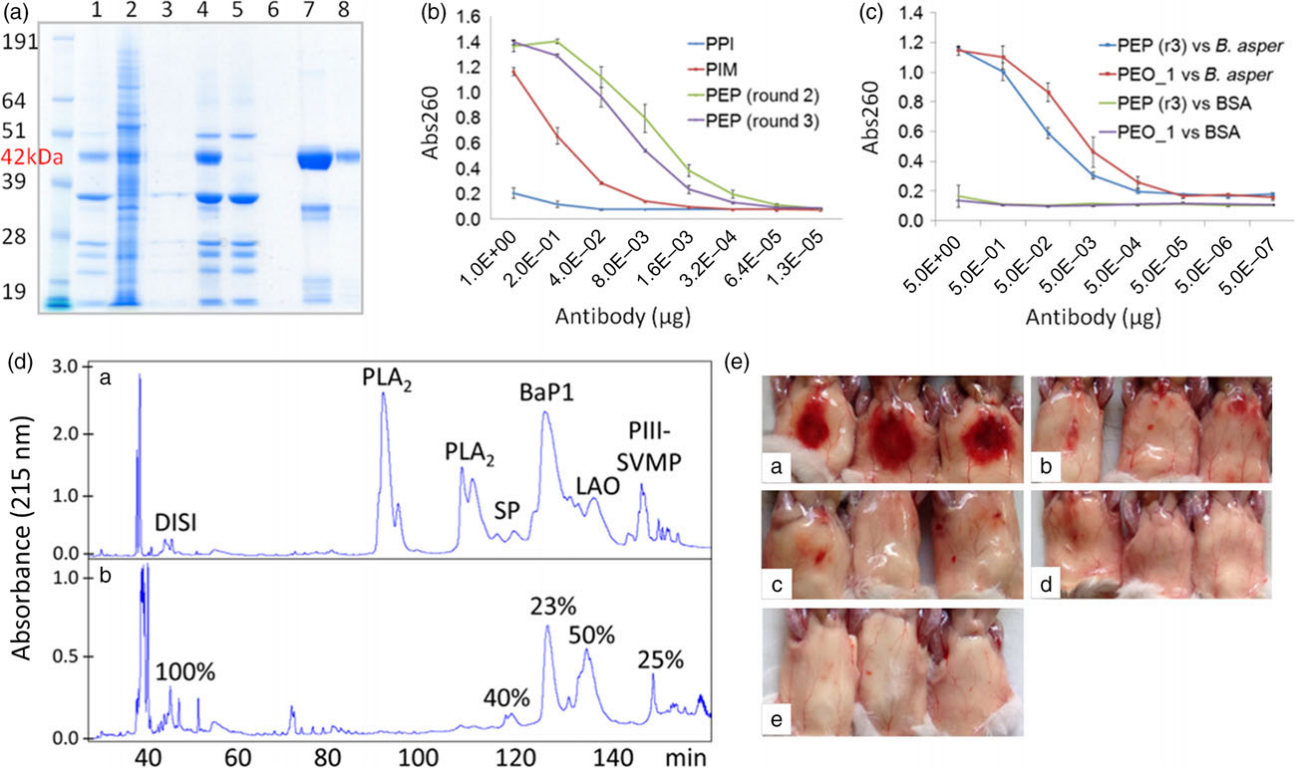
Assessment of plantivenom functionality (A) Coomassie gel of an enriched oligoclonal plantivenom (PEO_1) production and purification steps: lane1, crude apoplastic fluid; lane 2, leaf crude extract; lane 3 control apoplastic fluid from uninfected leaf; lane 4 clarified apoplastic fluid; lane 5, protein A flow through; line 6, first wash; lanes 7 and 8, eluted plantivenom. (B) Comparison of ELISA binding activities against *B. asper* venom of sequential plantivenom enrichment steps. PPI, pre‐immune plantivenom; PIM, immune plantivenom; PEP, enriched polyclonal plantivenom derived from second and third phage display selection rounds. (C) Comparison of binding activities between PEP and PEO_1. (D) Antivenomic profile of PEO_1 plantivenom. Upper (a) and lower (b) panels display, respectively, chromatographic profiles of whole Costa Rican *B. asper* (Pacific population) venom, and the venom fraction nonimmunoretained in the immobilized PEO_1 affinity column. (E) Neutralization of *B. aspe*r venom haemorrhagic activity *in vivo* with PEO_1 plantivenom preparation (46.5 mg protein/mL). The figure shows the abdominal surface of mouse skin after 2‐h injection with a constant dose of full venom and different amounts of plantivenom. (a) haemorrhagic spot appearing in mice injected intradermally with 15 μg (5 MHD) of venom; (b) inhibition of 5 MHD haemorrhagic spot with an antivenom dose of 250 μL PEO_1/mg venom; (c) 500 μL PEO_1/mg venom; (d) 750 μL PEO_1/mg venom; (e) negative control (plantivenom diluted in PBS).

In contrast to traditional antivenoms obtained from hyperimmune animal plasma, our pluribodies derive from PMBC pools. Hence, the genetic information retrieved only corresponds to a minor fraction of the reactive B cells made up of circulating memory cells on their way to the bone marrow. Therefore, in order to create a functional plant‐made antivenom (hereafter termed plantivenom), we decided to enrich our pluribody repertoires in toxin binders by introducing an intermediate *in vitro* selection step into the production flow chart (see Fig. S1). PCR‐amplified immune subrepertoires were first cloned in a phage display vector and then subjected to three successive rounds of selection against *B. asper* venom. To maximize diversity and avoid dominant single‐antigen selection bias, phage panning was conducted against different fractions of the venom (four in total, roughly corresponding to groups of toxin activities, Figure 4a). After selection, all four sublibraries were pooled and the variable antibody regions were amplified and transferred in bulk to the pGV_H_H_IgGH1 vector (Figure 4b) carrying the constant regions of human IgGH1 to create an enriched polyclonal (EP) master cell bank (MCB) with increased reactivity and potentially less likely to produce hypersensitivity reactions. EP_MCB was subsequently distributed in WCBs and agroinfiltrated in *N. benthamiana*. The PEP_1 formulation showed a 2xlog increase in total binding activity when compared with the nonenriched pluribody in ELISA (Figure 5A), demonstrating that enrichments in polyclonal compositions obtained by *in vitro* selection can be successfully transferred to the plant, where they globally reproduce the reactivity gains.

Finally, in order to produce the first iteration for a plantivenom candidate, we decided to produce a pluribody incorporating 36 individual clones that showed maximum binding activities. The clone composition of the resulting enriched oligoclonal formulation (EO_1, see Fig. S1) is detailed as supplementary information (Table S1). An EO_WCB was employed to scale up antibody production using one kilogram of agroinfiltrated *N. benthamiana* leaves and yielding 0.2 g of recombinant polyclonal antibody collected from the corresponding leaf apoplastic fluid. Following affinity purification (Figure 5A), an antivenom solution in PBS (PEO_1) was prepared at a concentration of 46.5 mg protein/mL, similar to the typical concentration of equivalent commercial antivenoms (55 mg/mL), and used for further characterization (summarized in Table 1). As expected, the PEO_1 solution showed enriched antivenom‐binding activity when assayed in ELISA against *B. asper* venom (Figure 5B, C). Figure 5D showed that phospholipases (PLA_2_), and to a lesser extent serine proteinases (SP), P‐I and P‐III snake venom metalloproteinases (SVMP) and L‐amino acid oxidase (LAO) molecules, were efficiently retained by the immobilized plantivenom. However, the affinity matrix was poor in capturing disintegrins (DISI). Such pattern of immunorecognition mirrors the immunological profile of monospecific (*B. asper*) antivenoms (Gutierrez *et al*., 2010), which were shown to quantitatively immunocapture most P‐III SVMPs, serine proteinases, L‐amino acid oxidase, but only to a lesser extent medium‐sized disintegrins, PLA_2_ molecules, some serine proteinases and P‐I SVMP BaP1. Later, the ability of PEO_1 to neutralize different *B. asper* toxic and enzymatic activities was analysed. The PEO_1 plantivenom was able to neutralize venom haemorrhagic, PLA_2_, proteinase and lethal activities (Table 1). Figure 5C illustrates the neutralization of haemorrhagic activity in the mouse skin test. The plantivenom did not neutralize *in vitro* coagulant activity on plasma at the maximum concentration tested (372 mg plantivenom/mg venom) (Table 1). Finally, we tested the capacity of PEO_1 to neutralize lethality induced by *B. asper* venom in mice, using 2 LD_50_s of venom as ‘challenge dose’. PEO_1 prevented lethality in all injected mice at a ratio of 61.24 mg plantivenom/mg venom (supporting information) with an estimated median effective dose (ED_50_) of 43 mg plantivenom/mg venom (95% confidence limits: 33–56 mg/mg) (Table 1). All PEO_1 neutralization activities were assayed in parallel with a commercial horse‐derived antivenom, and the corresponding neutralizing activities are also summarized in Table 1. Horse‐derived antivenom was effective in the neutralization of all enzymatic and toxic effects assayed.

**Table 1:** Neutralization of toxic activities of *B. asper* venom by plantivenom and equine‐derived antivenom

Toxic and enzymatic activities of *B. asper* venom	ED_50_ Plantivenom (mg/mg venom)	ED_50_ Equine‐derived (mg/mg venom)
Haemorrhagic	22.2 ± 0.65	6.9 ± 0.01
Phospholipase A_2_	224.1 ± 13.2	37.4 ± 0.4
Proteolytic	120.5 ± 4.2	43.0 ± 2.0
Peritoneal lethality	43.2 (33.4–56.0)	3.1 (1.2–8.4)
Coagulant on human plasma	≫372	11.3 ± 0.13

Neutralization of haemorrhagic, phospholipase A_2_, proteinase and lethal activities is expressed as median effective dose (ED_50_), whereas neutralization of in vitro coagulant activity is expressed as effective dose (ED), in terms of mg plantivenom or antivenom/mg venom protein (see Materials and methods for details). Results are presented as mean ± SD (*n* = 3) for neutralization of haemorrhagic, phospholipase A_2_, proteinase and coagulant effects. In the case of neutralization of lethal effect, the 95% confidence limits are included in parenthesis.

## Discussion

Global antivenom shortage has prompted several lines of research aimed at finding solutions to the main production bottlenecks. The use of immunization with DNA or with recombinant‐expressed epitopes of relevant toxins is an example of this, aimed at circumventing the shortage of antigens in species with low venom yield, such as coral snakes (Ramos *et al*., 2016). In comparison, fully recombinant strategies can provide a more satisfactory solution in the long term, addressing not only the shortage of antigens, but also other problems arising from current therapies such as adverse reactions and reproducibility (Laustsen *et al*., 2016). In theory, rationally designed cocktail formulations made of recombinant mAbs could outperform animal plasma‐derived antivenoms, as the composition of the latter comprises a large fraction (≥80%) of immunologically irrelevant antitoxin antibodies. In contrast, by adjusting the antivenom composition to a minimal number of selected functional components, the safety profile can be improved and manufacturing costs can be reduced. Minimal recombinant antibody cocktails comprising only three mAbs antibodies against the main *Bothrops* toxins were shown to neutralize the lethal toxicity of the venom (Frauches *et al*., 2013), indicating that oligoclonal cocktails are suitable antivenom formulations at least in some cases. Notwithstanding, a potent cocktail would probably require more complex combinations, incorporating antibodies against minor toxins, which could act alone or synergistically with other toxins, to ensure the coverage of intra‐ and interspecific variation. Complex cocktail composition imposes serious constraints on current manufacturing systems. Indeed, whereas the formulation of improved cocktails seems an accessible objective with current antibody selection techniques, the lack of suitable production systems for these combinations has proved a practical limitation, discouraging further advances. The pluribody strategy is in line with other recent strategies based on mammalian cell cultures aimed to produce affordable antibody cocktails (Laustsen, 2016; Rasmussen *et al*., 2012), which can foster the development of improved recombinant formulations. The employ of plant virus‐based expression systems offers further advantages associated with the use of plants as biofactories. As an example, the deconstructed virus strategy (Marillonnet *et al*., 2004, 2005) was successfully used to produce the Z‐Mapp anti‐Ebola protective antibody cocktail (Qiu *et al*., 2014) among other examples. Record yields up to 4.8 g/kg FW of anti‐idiotype antibodies have been reported using magnICON (Bendandi *et al*., 2010). With our current, nonoptimized yields, a 1000 sqm greenhouse facility growing 15‐kg biomass/m^2^/year (Walwyn *et al*., 2015) could produce up to 3000 1.0 gram doses per year. Further improvements in yield and formulation could reach 15 doses/m^2^/year as a reasonable scenario.

The synthetic diversity offered by somatic mosaicism can be exploited to quickly transfer ‘*in mass’* large immune mammalian subrepertoires, as exemplified with the PIM formulations. Although faithful reproduction of the mammalian repertoire cannot be guaranteed, we shown here that the antivenom‐binding profiles of large plant‐made repertoires largely resemble that of their corresponding antisera, and the same occurs during the successive *in vitro* enrichment steps, indicating that any possible expression bias does not affect substantially the binding profile of the final product. In the future, sampling of lymphoid organs and/or cell‐sorting procedures could be incorporated to enrich the composition of polyclonal mixtures. Alternatively, additional *in vitro* selection steps as those shown here can be used to refine composition, producing optimized oligoclonal mixtures. In this line, the decision to conduct our final efficacy tests with an oligoclonal formulation was taken with a double objective: first to maximize diversity, as for some antigens late phage display rounds could end up selecting strong monoclonal binders only and second to facilitate the dissection of the neutralization results, as the individual characterization of the antibodies in the cocktail will facilitate improvements in subsequent formulations via incorporation of new (and distinct) individual clones.

The PEO_1 cocktail described here showed a reasonable spectrum of neutralizing activities with relatively little effort in antibody selection; however, further efforts will be required to reach efficacy levels of current commercial antivenoms. Envenomings by *Bothrops asper* are characterized by several pathological and pathophysiological alterations, among which alterations in the coagulation are important. Thus, any antivenom to be considered for use in envenomings by *B. asper* should be able to neutralize the coagulant effect of the venom. This should prompt further efforts to increase the neutralizing capacity of the plantivenom and to generate neutralization of the coagulant activity. With consistency and reproducibility ensured with the pluribody strategy, the opportunities for formulation improvements through the implementation of successive design–build–test cycles are manifold. The neutralization of coagulant activity can be improved with the introduction of new serine protease neutralizing clones from improved antibody selection procedures, synthetic repertoires or alternative single‐cell selection methods (Beerli and Rader, 2010). A streamlined formulation should also involve the elimination of clones with overlapping or non‐neutralizing activities and, ultimately, the incorporation of neutralizing activities against other snake venoms from the same region. Our results should be followed by a more in depth preclinical assessment of the plantivenom, using various animal and *in vitro* models. Once a plantivenom with the capacity to neutralize all toxic activities of the venom is developed, the next step would be to consider the implementation of clinical trials and to stablish clinical doses. The dose of antivenom to be administered in clinical cases cannot be directly extrapolated from the preclinical neutralization tests. It has been roughly estimated that an adult *B. asper* specimen may inject 50 mg of venom in a bite, although this varies from bite to bite according to many variables (size of the snake, volume of venom available in the gland, strength of the bite, etc.). Once an antivenom has demonstrated efficacy in preclinical tests, clinical trials involve an initial dose‐finding phase, in which the optimal dose of antivenom is selected. This dose is then used for more extensive clinical trials with a higher number of patients.

The manipulation of SE for manufacturing purposes is pivotal in the pluribody approach. SE ensures the independence of each micro‐production line, resulting in highly reproducible final products. The resulting structured population distribution simplifies the modelling of the production process, as the whole system can be regarded as the sum of thousands of independent micro‐production lines (expression tiles) working in parallel. This work also underscores the potential of plant synthetic biology to offer the world new bio‐fabrication solutions. Pluribody technology combines genetic elements from biological sources as distant as camels, humans and plant viruses to capture the immune repertoire of hyperimmunized camels and recapitulate this within the plant chassis, creating plantivenoms able to neutralize a wide range of toxin activities that provide protection against lethal venom doses in mice. Beyond antivenoms, we believe that the ability to formulate pluribodies *à la carte* could provide a new boost to passive immunotherapy, facilitating the formulation and manufacturing of new, affordable antitoxin and antimicrobial products.

## Materials and methods

Detailed information about the experimental methods can be found in SI. All procedures involving experimental mice meet the requirements of the Guiding Principles for Biomedical Research Involving Animals (CIOMS, 1985) and were approved by the Institutional Committee for the Care and Use of Laboratory Animals of Universidad de Costa Rica (approval number 82‐08). The camel immunization protocol followed animal experimentation guidelines published by the regional government of the Canary Islands (Spain) and was approved by the Ethics Committee, Veterinary Medicine Service, Las Palmas de Gran Canaria University Foundation (Ref.: 009/2011)

### Snake venoms and venom fractions

Venoms were obtained from specimens of *B. asper*,* C. scutulatus scutulatus* and *C. simus* at the Serpentarium of the Instituto Clodomiro Picado (University of Costa Rica, San José). For venom fractionation, *B. asper* venom proteins were separated by reverse‐phase HPLC; the resolved chromatographic peaks (48) were collected separately and the eluted fractions pooled in four groups: group 1 was mainly composed of disintegrins; group 2 of phospholipases; group 3 mainly contained serine proteases and PLA2; and group 4 was enriched in metalloproteinases.

### Camel immunization, total RNA isolation and amplification of V_H_H sequences

Three camels (*Camelus dromedarius*) were immunized with a cocktail containing equal amount of each of three snake venoms (*C. scutulatus*,* C. simus* and *B. asper*). Peripheral blood lymphocytes (PBLs) obtained from ‘pre‐immune’ and ‘immune’ samples were used to isolate RNA and synthesize cDNA to be used as template for PCR amplification of V_H_H coding sequences (see supplementary materials and methods and Table S2 for the list of primers used).

### V_H_H Phage Display library construction and selection

The amplified V_H_H sequences were directly cloned as PCR products into the pSword phagemid vector and the ligation products transformed into *E. coli*. In a first round, venom binders were selected using *B. asper* full venom as the antigen. Subsequent selection rounds were carried out separately against each one of the four antigen groups obtained by venom fractionation. Monitoring of the library enrichment was carried out by polyclonal phage ELISA on *B. asper* full venom. Monoclonal phage ELISA was carried out in the same way from individual clones of the third round of selection; clones showing the highest binding affinity for each individual antigen were selected to formulate an oligoclonal mix: two clones from group 1; eight from group 2; six from group 3; and 20 from group 4 (SI).

### V_H_H plant expression libraries

V_H_H sequences amplified from PBL cDNA were directly cloned as PCR products into pGV_H_H‐His. Selected V_H_H phage display libraries and individual clones were cloned in pGV_H_H‐IgG vector. pGV_H_H‐His and pGV_H_H‐IgG are adaptation of the magnICON plant viral expression vector pICH7410 (ICON Genetics) built on a pDGB1 vector backbone (Sarrion‐Perdigones et al., 2011). Ligation products were transformed into *E. coli* cells and plated on solid LB agar plates. For libraries, bacteria were scraped and stored in 1 mL aliquots as *E. coli* library stocks. One‐tenth of the library was used for plasmid DNA isolation. For individual clones, a single colony was used to inoculate a fresh culture that was afterwards used for plasmid DNA isolation, the remaining culture being stored in 1 mL aliquots as *E. coli* glycerol stocks. The isolated plasmid DNA was transformed into the *Agrobacterium tumefaciens* GV3101 strain. The transformation was spread on LB plates. Bacteria were scraped and stored in 500 μL aliquots as library stocks to create a master cell bank (MCB). MCB aliquots were grown O/N and distributed in working cell banks (WCB) used for agroinfiltration.

### Estimation of the polyclonal diversity of the somatic mosaic

To determine average tile size in a typical polyclonal experiment, a pGV_H_H‐His antibody library was mixed with a pGGFP clone in different ratios (1%, 5% and 10%) and co‐infiltrated at different OD600s (0.1, 0.03, 0.01 and 0.003) in *N. benthamiana* leaves. Images of each experimental point taken at days 5 and 10 p.i were analysed with ImageJ software (http://rsb.info.nih.gov/ij/), and the number of tiles and average tile size were calculated using the ‘Analyse particle’ function. An experimental surface/weight conversion rate of 35.2 cm^2^/g FW in *N. benthamiana* leaves was applied to calculate the number of expression tiles per biomass.

### Transient expression in *Nicotiana benthamiana*

For polyclonal and oligoclonal agroinfiltrations, an aliquot of the WCB collection was used to inoculate an appropriate volume of LB supplemented with antibiotics (1 : 250 subculture); cultures were grown for 8 h at 28 °C. Cultures were collected by centrifugation (10 min, 3000 ***g***), re‐suspended in agroinfiltration medium (10 mm MES pH 5.6, 10 mm MgCl_2_, 200 μm acetosyringone) to an optical density at 600 nm of 0.05 and incubated for 2 h at room temperature on a horizontal rolling mixer. Before infiltration, the bacterial suspensions harbouring V_H_H‐6xHis or V_H_H‐Fc (3′‐provector modules) were mixed with equal volumes of the pICH14011 (Integrase) and pICH17388 (5′‐provector module) suspensions. For agroinfiltration, 5‐ to 6‐week‐old *N. benthamiana* plants were submerged in the agroinfiltration solution, a vacuum was applied to a pressure of −0.9 bar (Vacuum Degassing Chamber DP118, Applied Vacuum Engineering, Thornbury, UK), held for 1 min and then slowly released. Samples for protein extraction were collected 7–10 days postinfiltration.

### Analysis of diversity and reproducibility at DNA level

Sequencing libraries were prepared from V_H_H‐6xHis immune and pre‐immune library agroinfiltrated *N. benthamiana* leaves (see supplementary materials and methods and Table S2 for the list of primers used). Libraries were sequenced on an Ion Torrent PGM at Life Sequencing (Valencia, Spain). Sequence quality trimming, de novo assembly and posterior analysis were conducted using custom Python scripts and iAssembler software (Zheng *et al*., 2011). Unigenes composed of at least two members were used for library comparison by BLASTN using the Blast User DB tool at http://nbc11.biologie.uni-kl.de/.

### Computer simulation of Somatic expression mosaics

Simulation of expression mosaic formation was performed with NetLogo 6.0 agent‐based modelling software (Wilensky, 1999), with a script designed to simulate cell‐to‐cell movement of exclusion‐enabled viral clones on two‐dimensional hexagonal grid (Wilensky, 2007) (http://pgb.ibmcp.csic.es/netlogo/netlogo.html and SI).

### Antivenomics

Affinity chromatography‐based antivenomic (Pla *et al*., 2012) was employed to address the immunocapturing ability of PEO_1 towards *B. asper* venom components. Columns were prepared by immobilizing plantivenom on cyanogen bromide‐activated Sepharose 6 MB (SIGMA, St. Louis, USA), following the manufacturer's instructions. The column was then loaded with *B. asper* venom pooled from *B. asper* specimens from the Pacific versant of Costa Rica. Bound and nonretained fractions were analysed by HPLC. Chromatographic runs of whole *B. asper* venom dissolved in PBS were used as controls.

### Neutralization of toxic activities

The ability of plantivenom to neutralize proteinase, phospholipase A2 (PLA2), hemorrhagic, coagulant, and lethal activities was determined as previously described (Bolanos, 1972; Gene et al., 1989; Gutierrez et al., 1985; Gutierrez et al., 1986; Gutierrez et al., 2008; Theakston and Reid, 1983; Wang et al., 2004). Neutralization was expressed as median effective dose (ED_50_) for lethal, haemorrhagic, proteinase and PLA_2_ activities (plantivenom/venom, or equine antivenom/venom, ratio in which the effect was inhibited by 50% (Segura *et al*., 2010)), or as effective dose (ED) for coagulant activity (the plantivenom/venom, or equine antivenom/venom, ratio in which the clotting time was prolonged three times as compared to venom incubated with PBS instead of antibodies (Gene *et al*., 1989).

## Supporting information


**Figure S1** Schema for the production of different pluribody formulations representing camel antibody subrepertoires.
**Table S1** Characterization of individual clones comprising PEO_1 plantivenom. Binding activities against venom and compared with BSA are shown next to the amino acid sequence.
**Table S2** Primers used for VHH cloning and generation of sequencing libraries.
