# Supplementary material for: A synthetic biology approach for consistent production of plant‐made recombinant polyclonal antibodies against snake venom toxins
Source: Plant Biotechnol J. 2017 Sep 22;16(3):727–36. doi: 10.1111/pbi.12823 (PMC5814581; doi:10.1111/pbi.12823)
Supplement: Supplementary file 1 — Figure S1 Schema for the production of different pluribody formulations representing camel antibody subrepertoires. Table S1 Characterization of individual clones comprising PEO_1 plantivenom. Binding activities against venom and compared with BSA are shown next to the amino acid sequence. Table S2 Primers used for VHH cloning and generation of sequencing libraries. [file PBI-16-727-s001.docx]

**SUPPLEMENTARY INFORMATION**

**MATERIALS AND METHODS**

**Snake venoms and venom fractions**

Venoms were obtained by manual extraction (milking) from specimens of *B. asper*, *C. scutulatus scutulatus* and *C. simus* kept in captivity at the Serpentarium of the Instituto Clodomiro Picado (University of Costa Rica, San José). After extraction, the venoms were lyophilized and stored at −20 °C until used. For venom fractionation, 2 mg of lyophilized *B. asper* venom proteins were separated by reverse-phase HPLC and the resolved chromatographic peaks (48) were collected separately. Eluted fractions were then pooled in four groups. Group 1 was mainly composed of disintegrins; group 2 of phospholipases; group 3 mainly contained serine proteases and PLA2; and group 4 was a heterogeneous group enriched in metalloproteinases.

**Camel immunization, total RNA isolation, and amplification of V_H_H sequences**

The camel immunization protocol followed animal experimentation guidelines published by the regional government of the Canary Islands (Spain) and was approved by the Ethics Committee, Veterinary Medicine Service, Las Palmas de Gran Canaria University Foundation (Ref.: 009/2011). Three camels (*Camelus dromedarius*) were immunized with a cocktail containing equal amount of each of three snake venoms (*C. scutulatus*, *C. simus*, and *B. asper*) mixed with Freund adjuvant in a 1:1 ratio (v/v). The camel immunization protocol followed animal experimentation guidelines published by the regional government of the Canary Islands (Spain) and was approved by the Ethics Committee, Veterinary Medicine Service, Las Palmas de Gran Canaria University Foundation (Ref.: 009/2011). Before immunization, 150 mL citrated "pre-immune" blood was collected from the jugular vein for the isolation of lymphocytes. The animals were injected subcutaneously. Boosts were given at one week intervals for 5 consecutive weeks. One week after the last boost, anticoagulated "immune" blood was collected as described above. Peripheral blood lymphocytes (PBLs) were obtained from the "pre-immune" and "immune" samples using the Ficoll-Paque density gradient (GE Healthcare, Little Chalfont, UK) according to the manufacturer´s instructions. Plasma from both samples was also collected to evaluate the animals’ immune response against the antigens. Total RNA was isolated from PBLs using TRIzol® reagent (Invitrogen, Life Technologies, Paisley, UK) as described in the product manual. Synthesis of cDNA was carried out with oligo-d(T) primer using the Superscript® First Strand Synthesis System for RT-PCR (Invitrogen, Life Technologies, Paisley, UK) following the recommended protocol for GC-rich templates. The cDNA was used as a template for the amplification of the V_H_H coding sequences in a nested PCR reaction using Phusion HF DNA polymerase (New England BioLabs, Ipswich, MA, USA). First, CALL001 and CALL002 primers (Saerens et al., 2004) (Table S2) were used to amplify the V_H_H-C_H_2 and V_H_-C_H_1-C_H_2 regions of heavy chain and conventional IgG antibodies, respectively. The V_H_H-C_H_2 amplification products were gel-purified using the GFX PCR DNA and Gel Band purification Kit (GE Healthcare, Little Chalfont, UK) and used as a template in a second PCR reaction with the V_H_H specific primers J12Mar01 and J12Mar02 or J13Oct01 and J13Oct02 (Table S2) to enable cloning in the plant expression pGVHH-His vector or the phagemid pSword vector, respectively.

**V_H_H Phage Display library construction and selection.**

Vector pSword derives from the pHEN-2 phagemid vector. The new vector incorporates a cloning cassette containing the CcdB and CamR selection genes flanked by BbsI cloning sites to allow in-frame fusion of the V_H_H sequences to the minor coat protein pIII. Additionally, the cassette incorporates BsaI sites to allow easy transfer of the cloned V_H_H sequences to plant expression vectors pGV_H_H-His and pGV_H_H-IgG. V_H_H sequences amplified from peripheral blood lymphocytes cDNA with primers J13Oct01 and J13Oct02 were directly cloned as PCR products into the pSword vector in a BbsI (Thermo Scientific, Waltham, MA, USA) golden gate restriction/ligation reaction (50x [37°C-2 min, 16°C-5 min]; 65°C-20 min). T4 DNA ligase was purchased from Promega (Wisconsin, USA); the reaction was carried out in T4 DNA ligase buffer supplemented with 10mM NaCl and BSA (0.1 mg/mL). After phenol-chloroform extraction followed by ethanol precipitation, ligation products were transformed into *E. coli* XL1 Blue MRF' cells (Stratagene, CA, USA) by electroporation and plated on solid LB agar plates (containing 12.5 µg/ml tetracycline, 50 µg/ml carbenicillin, 1% glucose). Tenfold serial dilutions of the transformation were plated in parallel for library titration. After o/n growth at 37°C, bacteria were scraped in LB medium containing 15% glycerol and stored in 1 mL aliquots at -80°C as library stocks. Phages were produced by infecting the bacterial library with VCS-M13 helper phage at a multiplicity of infection of 20 following standard procedures. In a first round, venom binders were selected separately for each of the three immune libraries generated using *B. asper* full venom as the antigen. Aliquots of 1st round enriched libraries were then pooled and used for subsequent, more stringent selection rounds against each one of the 4 antigen groups obtained by venom fractionation. Binders were selected in Costar 96 Well EIA/RIA plates (Corning, NY, USA) according to standard procedures. Monitoring of the library enrichment was carried out by polyclonal phage ELISA on *B. asper* full venom (2 µg/well). Anti-fd bacteriophage (SIGMA, St. Louis, USA) was used as the primary antibody at a 1:1000 dilution; ECL^TM^ peroxidase labelled anti-rabbit IgG antibody (GE Healthcare, Buckinghamshire, UK) at a 1:5000 dilution was used as the secondary antibody. Detection was performed with o-phenylenediamine dihydrochloride substrate (SIGMAFAST™ OPD tablet, SIGMA, St. Louis, USA). Reactions were stopped with 3.0 M HCl. Absorbance was determined at 492 nm. To control for binding specificity, plates were coated with 10 μg/mL of BSA. Additionally, monoclonal phage ELISA was carried out in the same way from individual clones of the 3rd round of selection; clones showing the highest binding affinity for each individual antigen were selected to formulate an oligoclonal mix: 2 clones from group 1; 8 from group 2; 6 from group 3; and 20 from group 4 (Supplementary data).

**V_H_H plant expression libraries.**

Vector pGV_H_H-His is an adaptation of the magnICON plant viral expression vector pICH7410 (ICON Genetics). The T-DNA region of the vector was built in three different modules. The first module contained the attB site and the intron from pICH7419, and the tobacco (1-3)-beta-glucanase secretory pathway targeting signal peptide cloned in the pVD1 vector (www.gbcloning.upv.es). The second module contained a LacZ cassette flanked by BsaI sites cloned in pGEM-T Easy (Promega, Wisconsin, USA). The third module harbored a 6x-His-Tag, the cr-TMV 3′ non-translated region from pICH7410, and the nopaline synthase terminator (Tnos) cloned in pVD1. The three modules were assembled together with the modules conforming the pDGB1 vector backbone (Sarrion-Perdigones et al., 2011), to build pGV_H_H -His. Vector pGV_H_H-IgG is identical to pGV_H_H-His except for the 6x-His-Tag which is replaced by the Fc region of human IgG1. V_H_H sequences amplified from peripheral blood lymphocyte cDNA with primers J12Mar01 and J12Mar02 were directly cloned as PCR products into pGV_H_H-His vector in a BsaI (NEB, Ipswich, MA, USA) golden gate restriction/ligation reaction (50x [37°C-2 min, 16°C-5 min]; 65°C-20 min). Selected V_H_H phage display libraries and individual clones were cloned in pGV_H_H-IgG vector using the same restriction/ligation reaction. The ligation products were transformed into TOP10 *E. coli* cells by electroporation (V_H_H libraries) or heat-shock (individual clones) and plated on solid LB agar plates containing 50 µg/mL kanamycin. For libraries, bacteria were scraped in LB medium containing 50 µg/mL kanamycin and 15% glycerol and stored in 1 mL aliquots at -80°C as *E. coli* library stocks. One tenth of the library was grown for 2h at 37°C and used for plasmid DNA isolation. For individual clones, a single colony was used to inoculate a fresh culture that was afterwards used for plasmid DNA isolation; the remaining culture being stored at -80°C in 1 mL aliquots as *E. coli* glycerol stocks. The isolated plasmid DNA library stock was transformed into the *Agrobacterium tumefaciens* GV3101 strain (carrying the pSoup plasmid) by electroporation. The transformation was spread on LB plates containing 50 µg/mL kanamycin and 50 µg/mL rifampicin. Bacteria were scraped in LB medium containing 50 µg/mL kanamycin and 50 µg/mL rifampicin and 15% glycerol, and stored in 500 µL aliquots at -80°C as library stocks to create a Master Cell Bank (MCB). MCB aliquots were grown O/N and distributed in Working Cell Banks (WCB) used for agroinfiltration

**Extraction and Purification of V_H_H-6xHis and V_H_H-Fc antibodies from plant leaf tissue**

Recombinant proteins from agroinfiltrated plants were extracted from the leaf apoplast by vacuum infiltration-centrifugation. Leaves were excised from the plant, rinsed in distilled water, and blot dried on tissue paper. Afterwards, the leaves were submerged in cold extraction buffer; a vacuum was applied to a pressure of -0.9 bar (Vacuum Degassing Chamber DP118, Applied Vacuum Engineering, Thornbury, UK), held for 1 min, and then slowly released to allow the buffer to infiltrate the leaf blades. Na_2_HPO_4_ 40mM, 50mM ascorbic acid, EDTA 10mM, pH7 was used as the extraction buffer for the V_H_H-Fc expressing tissue; V_H_H-6xHis expressing tissue samples were extracted in 50mM NaH_2_PO_4_, 300mM NaCl, 10mM Imidazol, 25mM ascorbic acid, pH8. Vacuum infiltrated leaves were then blot dried on tissue paper and centrifuged in a swing-rotor centrifuge (10 min, 1200xg) at 4°C to extract the apoplast fluid (AF). The recombinant 6xHis-tag V_H_Hs was purified by Ni-NTA affinity chromatography following a batch/gravity-flow procedure. Protein extraction was conducted as described above. The AF was further clarified by filtration through a 0.22 µm membrane filter. For purification purposes, 1 mL of resin suspension (Chelating Low Density Nickel agarose resin from ABT, Madrid, Spain) was equilibrated with extraction buffer following the manufacturer’s instructions. The equilibrated resin was mixed with 10 mL of clarified plant apoplast extract on an end-over-end rotator for 10 min at 4°C. The sample-resin mix was then loaded into a Poly-Prep® chromatography column (Bio-Rad Laboratories, Hercules, CA, USA) and washed three times with 10-bead volumes of wash buffer (50 mM NaH_2_PO_4_, 300 mM NaCl, 20 mM imidazole, pH8). Bound proteins were eluted from the column with 3 mL elution buffer (50 mM NaH_2_PO_4_, pH4) collected in 1 mL fractions; each fraction was immediately neutralized with 200 µl 500 mM PBS pH7.2. V_H_H-Fc antibodies were purified by ammonium sulphate precipitation and affinity chromatography using protein A Sepharose affinity columns. Proteins in the AF were sequentially precipitated by adding ammonium sulphate at 20% and 60% saturation followed by incubation at 4°C for 2h and centrifugation at 10,000xg, 4°C, for 30 min. The pellet from the second precipitation was re-suspended in 1/5 the original volume of extraction buffer and dialyzed against binding buffer (20 mM phosphate, 10mM ascorbic acid, pH7). The sample was further clarified by filtration through a 0.22 µm membrane filter. Affinity purification was performed by FPLC using an ÄKTA purifier (GE Healthcare, Buckinghamshire, UK) equipped with a HiTrap Protein A HP affinity column (GE Healthcare, Buckinghamshire, UK) as recommended by the manufacturer. Samples were eluted with 0.1 M citric acid pH 3.0 and neutralized with 1.0 M Tris-HCl pH 9.0.

**Analysis of diversity and reproducibility at protein level**

Independent samples of purified V_H_H-6xHis pluribodies produced in *N. benthamiana* leaves and representing different WCB derived from pre-immune (PPI) and immune (PIM) libraries were analyzed by 2D DIGE. Protein precipitation, fluorescent labeling, 2D electrophoresis, and silver staining were performed as previously described (Munoz-Fambuena et al., 2013). Each 2D gel was loaded with three samples (PIM_1, PIM_2, and PIM_3 for gel 1 and PIM_1, PIM_2, PPI_1 for gel 2) labeled with a different CyDye (Cy2, Cy3, and Cy5). Gels were scanned using a Typhoon Trio (GE Healthcare, Buckinghamshire, UK). Gel image analysis was performed with DeCyder 2D software v. 6.5.

**Analysis of diversity and reproducibility at DNA level**

Sequencing libraries were prepared from approximately 2 g of V_H_H-6xHis immune and pre-immune library agroinfiltrated *N. benthamiana* leaves. DNase treatment was carried out with an Ambion DNA-free™ Kit (Thermo Fisher Scientific, Waltham, MA, USA) following the manufacturer’s instructions. We prepared cDNA with the Superscript® First Strand Synthesis System for RT-PCR (Invitrogen, Life Technologies, Paisley, UK) using a gene-specific primer (D09OCT02pICHR1, Table S2) annealing at the cr-TMV 3′ non-translated region. PCR amplification of the V_H_H sequences was performed with an array of four forward primers (one for each sample) and a single reverse primer (Table S2). The forward primers (Pre-Immune1_Fwd, Pre-Immune2_Fwd, Immune1_Fwd, Immune2_Fwd) anneal to the FR1 (framework region1) and incorporate a unique IonXpress barcode adapter at their 5' end; the reverse primer (IT_Rev) binds to the FR4 and incorporates the Ion Torrent P1 adaptor region at their 5' end. PCRs were performed with Platinum® PCR SuperMix High Fidelity (Invitrogen, Life Technologies, Paisley, UK). Amplification products were purified with the Agencourt AMPure XP Reagent (Beckman Coulter, Inc, CA, USA). Libraries were sequenced on an Ion Torrent PGM at Life Sequencing (Valencia, Spain). Sequence quality trimming, de novo assembly, and posterior analysis were conducted using custom Python scripts and iAssembler software (Zheng et al., 2011). Unigenes composed of at least 2 members were used for library comparison by BLASTN using the Blast User DB tool at <http://nbc11.biologie.uni-kl.de/>.

**ELISA evaluation of antibody binding activity against snake venom**

Costar 96 Well EIA/RIA plates (Corning, NY, USA) were coated (o/n, 4°C) with 20 µg/mL of whole venom in coating buffer (50 mM carbonate buffer pH 9.8). Blocking was performed for 2 h at RT in a 2% (w/v) solution of ECL Advance^TM^ Blocking Reagent (GE Healthcare, Buckinghamshire, UK) in PBS-T (20 mM NaH_2_PO_4_, 80 mM Na_2_HPO_4_, 100 mM NaCl, pH7.4, supplemented with 0.1% (v/v) Tween 20). Camel plasma or purified pluribody samples diluted in PBS (20 mM NaH_2_PO_4_, 80 mM Na_2_HPO_4_, 100 mM NaCl, pH7.4) were then added to the wells and incubated for 45 min at RT. After 4 washings, 1:2000 rabbit anti-human IgG (SIGMA, St. Louis, USA) in PBS-T was added and incubated for 1 h at room temperature. Plates were washed 4 times and incubated with ECL^TM^ peroxidase labeled anti-rabbit IgG antibody (GE Healthcare, Buckinghamshire, UK) at a 1:5000 dilution for 1 h at RT. After washing the plates, o-phenylenediamine dihydrochloride substrate (SIGMAFAST™ OPD tablet, SIGMA, St. Louis, USA) was added for detection; reactions were stopped with 3 M HCl. Absorbance was determined at 492 nm. To control for binding specificity, plates were coated with 10 μg/mL of BSA. All washing steps were performed with PBS.

**Computer simulation of Somatic expression mosaics**

Simulation of expression mosaic formation was performed with NetLogo 6.0 agent-based modeling software (Wilensky, 1999), with a script designed to simulate cell–to–cell movement of exclusion-enabled viral clones on two-dimensional surfaces (<http://pgb.ibmcp.csic.es/netlogo/netlogo.html> and Supplementary information). Briefly, a two-dimensional hexagonal grid (Wilensky, 2007) of 500 x 500 patches was defined, each patch representing a plant mesophyll cell and having six neighboring patches. The hexagon was chosen to simulate the reported average number of cells that can be infected from a given cell (6.5±0.006) (Gonzalez-Jara et al., 2009). The variables used where (i) the number of initial infection foci for each clone (Virtual OD_600_, VOD), and (ii) the cell–to–cell movement speed (Virtual Fitness, VF), a parameter considered to be recombinant protein/clone-dependent. The VOD of each clone is represented by the number of initial infection foci and can be adjusted through the GFP, BFP, and DsRed-number sliders. Based on experimental observations, we established VOD of 3000 and 300 as matching experimental ODs of 0.1 and 0.01, respectively for the working grid size. In each cycle, viral particles will only infect uninfected neighboring cells. Virtual fitness is a parameter in the script that determines the relative chances of each competing clone to infect a virus-free neighboring cell. Differences in fitness (VF) between clones can be adjusted using the fitness slider.

**Antivenomics**

Affinity chromatography-based antivenomic (Pla et al., 2012) was employed to address the immunocapturing ability of PEO_1 towards *B. asper* venom components. Affinity chromatography columns were prepared by immobilizing 16 mg of plantivenom on 10 mL of cyanogen bromide-activated Sepharose 6MB (Sigma; catalog Number C9267), following the manufacturer's instructions. Columns were balanced with PBS, and absorbance was recorded at 280 nm. Then, each column was loaded with *B. asper* venom pooled from *B. asper* specimens from the Pacific versant of Costa Rica and washed with PBS. The bound fraction was eluted by changing the mobile phase to glycine, pH 3.0, and the pH of the collected fractions was adjusted by the addition of 0.5 M NaOH to reach pH 7.0–7.5. Subsequently, the bound fractions and the non-retained fractions were analyzed by HPLC, using an Agilent Technologies 1100 series system (Santa Clara, CA, USA) equipped with a chromatographic data management system (ChemStation Data Analysis and Reporting, Agilent Technologies) and using a Lichrosphere RP 100 C18 column (250 × 4.6 mm, 5 μm particle size). Fractions were eluted at 1.0 mL/min with a linear gradient of buffer A (95% water, 0.1% TFA) and buffer B (95% acetonitrile, 0.1% TFA) (5% B for 10 min, followed by 5%–15% B over 20 min, 15%–45% B over 120 min, and 45%–70% B over 20 min). Detection was set at 215 nm. Chromatographic runs of whole *B. asper* venom dissolved in PBS were used as controls.

**Neutralization of toxic activities**

The ability of plantivenom to neutralize proteinase, phospholipase A_2_ (PLA_2_), hemorrhagic, coagulant, and lethal activities of *B. asper* venom was determined by following methodologies previously described. Proteinase activity was assessed using azocasein as substrate (Gutierrez et al., 2008; Wang et al., 2004). PLA_2_ activity was determined using egg yolk phospholipids (Gutierrez et al., 1986). Hemorrhagic activity was determined in CD-1 mice (18-20 g) according to Gutiérrez et al. (1985) (Gutierrez et al., 1985). In vitro coagulant activity of venom was assessed in human plasma obtained from healthy volunteers (Gene et al., 1989; Theakston and Reid, 1983). Lethal activity was determined by intraperitoneal (i.p.) injection (Bolanos, 1972). Mice of both sexes of the CD-1 strain, weighing 18-20 g, were used for the assessment of hemorrhagic and lethal activities. All procedures involving experimental animals meet the requirements of the Guiding Principles for Biomedical Research Involving Animals (CIOMS, 1985), and were approved by the Institutional Committee for the Care and Use of Laboratory Animals of Universidad de Costa Rica (approval number 82-08).

For assessing the neutralizing capacity of plantivenom, the protocols used by Gutiérrez et al. (2008) (Gutierrez et al., 2008) and Segura et al. (2010) (Segura et al., 2010) were followed. Basically, for each effect, a ‘challenge dose’ of venom, i.e. the dose of venom to be tested in the corresponding experimental systems, was selected, and incubated with various amounts of plantivenom protein for 30 min at 37 °C. The ‘challenge doses’ for toxic activities were the following: for hemorrhagic activity, five Minimum Hemorrhagic Doses (MHDs); for coagulant activity, two Minimum Coagulant Doses (MCDs); and for lethal activity, two Median Lethal Doses (LD_50_). For proteinase and PLA_2_ activities, the ‘challenge doses’ of venom used were 5 µg and 20 µg, respectively. Controls included venom incubated with PBS instead of plantivenom. For comparative purposes, experiments were also run by incubating venom with the equine polyvalent antivenom currently in use for the treatment of snakebite envenoming. This antivenom is produced at Instituto Clodomiro Picado (Universidad de Costa Rica) from the plasma of horses immunized with a mixture of the venoms of *B. asper*, *Crotalus simus* and *Lachesis stenophrys*. After incubation, aliquots of the mixtures were tested in the corresponding experimental assays described above. Neutralization was expressed as either Median Effective Dose (ED_50_) (for lethal, hemorrhagic, proteinase and PLA_2_ activities), defined as the plantivenom/venom, or equine antivenom/venom, ratio in which the effect was inhibited by 50% (Segura et al., 2010). In the case of coagulant activity, neutralization was expressed as Effective Dose (ED), corresponding to the plantivenom/venom, or equine antivenom/venom, ratio in which the clotting time was prolonged 3 times as compared to venom incubated with PBS instead of antibodies (Gene et al., 1989). A detailed account of the neutralization experiments involving the use of mice is included in the Supplementary text.

# DESCRIPTION OF THE METHODOLOGY FOLLOWED IN EXPERIMENTS INVOLVING THE USE OF ANIMALS

1. ***Rationale for the need to use animals in this study***

Part of this study included the assessment of the neutralizing ability of the mixture of recombinant antibodies, and an equine-derived antivenom, against some of the most relevant effects induced by the venom of the snake *Bothrops asper*. Although some of the assessments used only in vitro tests, and therefore did not require animals, such as ELISA tests, and determination of the neutralization of in vitro coagulant effect, and proteinase and phospholipase A_2_ enzymatic activities of the venom, it was necessary to study the neutralization of two in vivo activities, i.e. lethal and hemorrhagic effects. The study of the neutralization of lethal effect is the ‘gold standard’ in the evaluation of antivenom efficacy against a particular snake venom. Since the aim of this study was to demonstrate that the mixture of recombinant antibodies was effective against this venom, the assessment of neutralization of lethality was necessary to test the main hypothesis of the study. This test is performed in mice and the end-point is the survival of mice treated with a supra-lethal dose of venom. There is no valid surrogate test for the neutralization of lethality induced by snake venoms, and this therefore justifies the performance of this test in this study. Likewise, the study of the neutralization of hemorrhagic activity was determinant to judge the neutralizing ability of the recombinant antibody mixture. Hemorrhage is one of the most relevant effects induced by viperid snake venoms, such as the venom of *Bothrops asper*. Any antivenom aimed to treat envenomings by this species should be able to neutralize venom-induced hemorrhage. As in the case of lethality, there is no valid surrogate test that could substitute the use of mice in the assessment of hemorrhagic activity. Therefore, for a rigorous test of the main hypothesis of this study, i.e. that a mixture of recombinant antibodies generated in plants is effective in the neutralization of the venom of *B. asper*, animals tests to determine the neutralization of lethal and hemorrhagic activities were absolutely necessary to perform. In order to reduce animal suffering, experiments were design as to use the lowest possible number of mice.

1. ***Nature of ethical permissions to develop this study***

As stated in the manuscript, all procedures involving experimental animals meet the requirements of the Guiding Principles for Biomedical Research Involving Animals (CIOMS, 1985), and were approved by the Institutional Committee for the Care and Use of Laboratory Animals of Universidad de Costa Rica (CICUA) (approval number CICUA 82-08).

1. ***Details of the design of experiments involving the use of animals***
2. *Assay for assessing the neutralization of lethality*: The value of the Median Lethal Dose (LD_50_) of *B. asper* venom was that described by (Bolanos, 1972). For the determination of the neutralization of lethality by the recombinant antivenom, mixtures of a fixed amount of venom and variable concentrations of antibodies were prepared, in a constant final volume. After an incubation period of 30 min at 37 °C, aliquots of the mixtures were tested in groups of four mice of both sexes of the CD-1 strain, weighing 18-20 g. A control group was included in which the same concentration of venom was incubated with 0.14 M NaCl, 0.04 M phosphates, pH 7.2 (PBS), instead of antivenom. The aliquots of the mixtures of venom and antibodies, or venom and PBS, contained 4 LD_50_s of venom per mL. Aliquots of 0.5 mL were injected intraperitoneally, thus containing a ‘challenge dose’ of venom corresponding to 2 LD_50_s. In parallel with the determination of neutralization by the recombinant plant-derived antibodies, the ability of the equine polyvalent antivenom was also tested for comparative purposes, using an identical experimental protocol. In this case, three mice were used per each experimental group. After 48 hours , the number of dead mice was recorded, and the neutralizing ability of the antibody mixture was determined and expressed as the Median Effective Dose (ED_50_), i.e. the ratio mg venom per mg antibody protein at which 50% of the injected number of animals.. ED_50_ was estimated by using the Spearman-Karber method. In the neutralization assays, a total number of 9 groups of mice were used, involving a total number of 32 animals included in the assessment of the neutralization of recombinant plant-derived antibodies and of equine polyvalent antivenom. The design of these experiments followed experimental protocols used in previous studies which evaluated the neutralizing capacity of antivenoms (Gutierrez et al., 2008), (Segura et al., 2010).
3. *Assay for assessing the neutralization of hemorrhagic activity:* The value of the Minimum Hemorrhagic Dose of *B. asper* venom was that described by (Gutierrez et al., 1985). For the determination of the neutralizing ability of antibodies against hemorrhagic activity of venom, mixtures of a fixed amount of venom and variable concentrations of antibodies were prepared, in a constant final volume. After an incubation period of 30 min at 37 °C, aliquots of the mixtures were tested in groups of three mice having the same characteristics described above. A control group was included in which the same concentration of venom was incubated with PBS instead of antivenom. The aliquots of the mixtures of venom and antibodies, or venom and PBS, contained 5 MHDs of venom. In parallel with the determination of neutralization by the recombinant plant-derived antibodies, the ability of the equine polyvalent antivenom was also tested for comparative purposes using an identical experimental protocol. Aliquots of 0.1 mL of the mixtures were injected intradermally, in the ventral abdominal region, in groups of three CD-1 mice of both sexes (18-20 g). After 2 hours, mice were sacrificed by CO_2_ inhalation and the area of hemorrhagic lesions in the inner side of the skin was measured, as described above. Neutralization ability of antibodies was expressed as Median Effective Dose (ED_50_), defined as the ratio mg venom/mg antibody protein at which the diameter of the hemorrhagic lesion was reduced by 50% as compared to the lesion induced by venom incubated with PBS instead of antibodies (Gutierrez et al., 1985). In the neutralization assays, a total number of 4 groups of mice were used per antivenom, involving a total number of 24 animals. The design of these experiments followed experimental protocols used in previous studies which evaluated the neutralizing capacity of antivenoms (e.g. (Gutierrez et al., 2008),(Segura et al., 2010). No special equipments, with the exception of the balances required for weighing the mice and the venoms, were used for these neutralization studies, as the end results, i.e. death of animals or measurement of the hemorrhagic lesions, correspond to visual observations of the animals.

Animals were allocated randomly to each experimental group and placed in the cages. Once placed in the corresponding cages previously marked with the doses of venom, or the venom/antibodies mixtures, animals were injected in such a way that the time of injection of each experimental group was recorded in order to ensure that the indicated time of observation (48 hr in lethality tests, and 2 hr in hemorrhage tests) was followed.

1. *Other details concerning the use of mice*: Mice were provided by the Animal Colony Facility of Instituto Clodomiro Picado (University of Costa Rica). Before the experiments, animals were regularly subjected to Veterinary Medical care and surveillance, and animals used in the experiments were healthy and showed a normal growth curve during the weeks before the tests. Experiments were performed during day time (8 am to 4 pm). During experiments mice were kept in cages (length: 40 cm; width: 25 cm; height: 15 cm), and groups of three or four mice were used in each cage. Animals were kept with 5 cm of width of bedding material and were provided food (concentrated food for rodents) and water *ad libitum*, under a 12:12 hours light cycle. Before and during the experiments, animals were kept at a temperature range of 18-22 °C.
2. ***Results***

All mice used in the experiments, both in the estimation of LD_50_ and MHD, and in the estimation of the neutralizing ability (ED_50_), were in a weight range of 18-20 grams, and all were in good health conditions before the injection of venom or venom and antibody

1. Estimation of the intraperitoneal ED_50_ (neutralization of lethality) of recombinant plant-derived antibodies: The following table presents the data obtained in these experiments.

| Dose of recombinant plant-derived abs/*B. asper* venom  mg/mg | Animals Injected | Dead animals after 48 hours |
| --- | --- | --- |
| 26.28 | 4 | 4 |
| 34.87 | 4 | 3 |
| 46.5 | 4 | 2 |
| 61.84 | 4 | 0 |
| Snake venom control | 4 | 4 |

1. Estimation of the intraperitoneal ED_50_ (neutralization of lethality) of equine-derived snake antivenom: The following table presents the data obtained in these experiments.

| Dose of equine-derived snake antivenom/*B. asper* venom  mg/mg | Animals Injected | Dead animals after 48 hours |
| --- | --- | --- |
| 1.74 | 3 | 3 |
| 3.47 | 3 | 1 |
| 6.94 | 3 | 0 |
| Snake venom control | 3 | 3 |

(c) Estimation of the ED_50_ (neutralization of hemorrhagic activity) of recombinant plant-derived antibodies: The following table present the data obtained in these experiments.

| Dose of recombinant  plant-derived abs/*B. asper* venom  mg/mg | Animals Injected | Hemorrhagic area  (mm2) |
| --- | --- | --- |
| 11.62 | 3 | 99-59-100 |
| 23.25 | 3 | 27-68-76 |
| 34.87 | 3 | 0-0-12 |
| Snake venom control | 3 | 194-199-162 |

1. Estimation of the ED_50_ (neutralization of hemorrhagic activity) of equine-derived snake antivenom: The following table present the data obtained in these experiments.

| Dose of equine-derived snake antivenom/*B. asper* venom  mg/mg | Animals Injected | Hemorrhagic areaa  (mm2) |
| --- | --- | --- |
| 3.47 | 3 | 172-155-183 |
| 6.94 | 3 | 0-0-0 |
| 13.87 | 3 | 0-0-0 |
| Snake venom control | 3 | 194-199-162 |

1. Adverse events: Since animals were treated with snake venom, or with venom/antibody mixtures, animals showed the typical signs of envenoming after injection of the venom, i.e. lethargy, reduced movement. These adverse events are part of the experimental setting and, therefore, no efforts were performed to reduce adverse events, as they are part of the experimental outcome of the experiments.
2. ***Limitations of the animal model used***

In experimental Toxinology, the two tests utilized, i.e. determination of lethal activity and determination of hemorrhagic activity, are widely used and accepted as tests to assess the toxicity of snake venoms. In the case of hemorrhage, the hemorrhagic lesions that develop in the skin of mice bear a close correlation with the hemorrhagic activity of venoms in experimental animals and in humans, since this effect is essentially due to the action of zinc-dependent metalloproteinases in the integrity of capillary vessel basement membranes. On the other hand, the lethality assay in mice is the ‘gold standard’ for the assessment of toxicity of snake venoms. The correlation between this lethality test between mice and humans is not clear, as it is likely that these two species have a different susceptibility to the action of lethal venom components. Thus, no quantitative extrapolation can be made between the doses required to kill a mouse and the doses required to kill humans. Nevertheless, this is a test that is routinely used for the assessment of venom toxicity and for testing the ability of antibodies to neutralize venom toxicity. Hence, an antibody, or an antibody mixture (such as antivenom) which is able to neutralize lethality of a particular venom in a mouse model is also able to block the lethal activity of this venom in humans. This has been repeatedly shown in the clinical setting with many venoms and antivenoms. Therefore, the two tests used in this study allow for a rigorous assessment of the ability of antibodies to neutralize these relevant toxic effects (lethality and hemorrhage) of the venom of *B. asper*.

1. ***Implications of the experimental design for the replacement, refinement or reduction /the 3Rs) of the use of animals in research***

There are not satisfactory tests that replace for the evaluation of lethal and hemorrhagic activities of snake venoms. In this regard, no possible replacement exists for the experiments designed to assess lethal and hemorrhagic activity of the venom and their neutralization by antibodies. The experimental design was prepared as to use the lowest number of animals while maintaining statistical significance of the results. In addition to these tests using mice, the ability of antibodies and antivenom to neutralize this venom was also assessed by using three different in vitro tests which do not require the use of mice. These are the ELISA for assessing the antibody titer and the neutralization of three relevant enzymatic and toxic activities, i.e. in vitro coagulant activity on plasma, phospholipase A_2_ activity, and proteinase activity. These activities are relevant because they bear a close relationship with some toxic activities of the venom. Thus, by using these *in vitro* tests, we reduced the number of mice and replaced *in vivo* tests and expanded the analysis of the neutralizing ability of the antibodies.

**NETLOGO SIMULATION**

Link to the three-clones simulation script:

<http://pgb.ibmcp.csic.es/netlogo/netlogo.html>

Instructions to run the program locally

1. Install Netlogo 6.0 locally. Download from <https://ccl.northwestern.edu/netlogo/download.shtml>
2. Download the simulation model at <http://pgb.ibmcp.csic.es/netlogo/netlogo.html> and run it locally with NetLogo:

i) Set the slider parameters to the desired values

GFP/BFP/DsRed-number: initial number of infection foci

GFP/BFP/DsRed-fitness: cell-to-cell movement speed

ii) Press the Set up button

iii) Press the Go button to start the simulation

**SUPPLEMETARY FIGURES**

Fig S1.

**Fig. S1. Schema for the production of different pluribody formulations representing camel antibody sub-repertoires.** Non-immunized PBMC samples were used to produce pre-immune pluribody preparations (PPI). PBMC from immunized camels were used directly to prepare immune polyclonal libraries in Agrobacterium, which were subsequently used to agroinfiltrate plants and produce immunized (PIM) pluribody preparations. The same immunized PBMC samples, subjected to an intermediate in vitro enrichment step via phage display, were used to generate either enriched polyclonal (PEP) or enriched oligoclonal (PEO) pluribody preparations. MCB, Agrobacterium master cell bank; WCB, Agrobacterium working cell bank; PI, pre-immune; IM, hyperimmune; EP, enriched polyclonal; EO, enriched oligoclonal.

**SUPPLEMETARY TABLES**

Table S1.


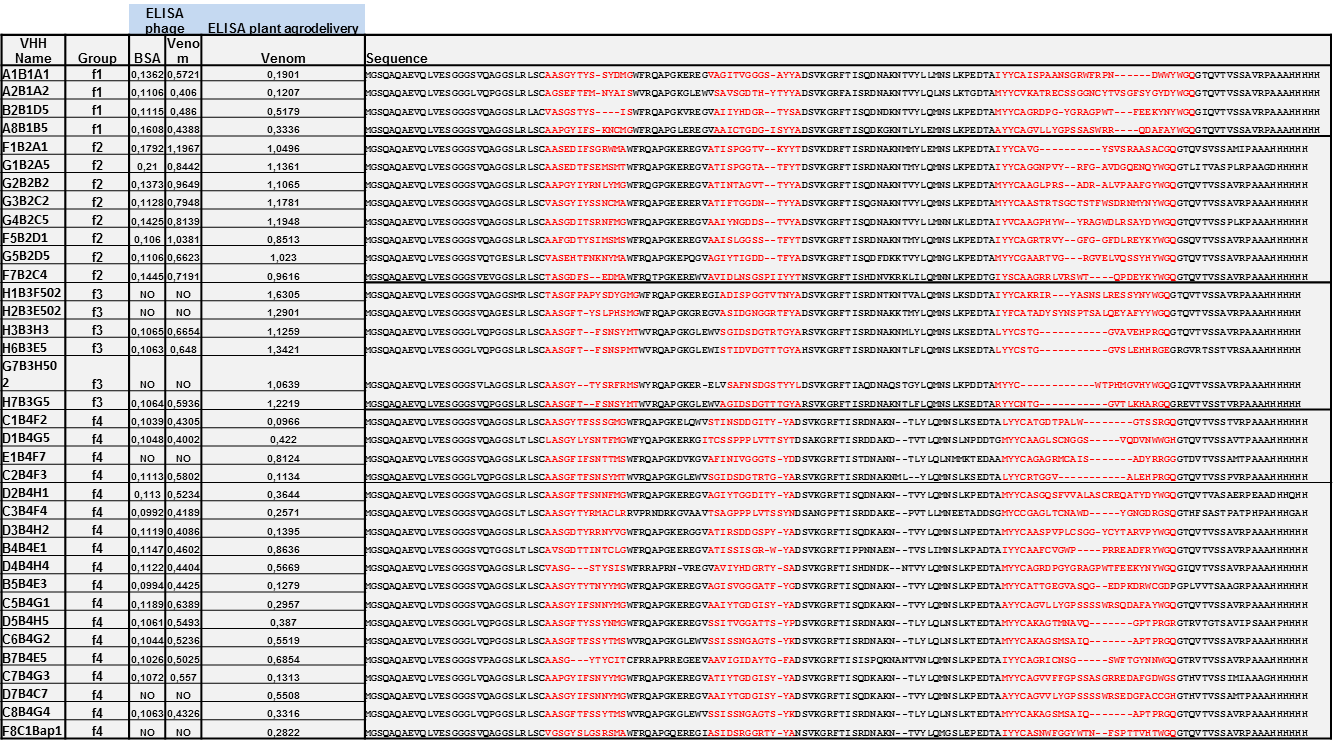
Characterization of individual clones comprising PEO_1 plantivenom. Binding activities against venom and compared with BSA are shown next to the amino-acid sequence.

Table S2.

Primers used for VHH cloning and generation of sequencing libraries

| Primer | Sequence |
| --- | --- |
| CALL001 | 5'-gtcctggctctcttctacaagg-3′ |
| CALL002 | 5′-ggtacgtgctgttgaactgttcc-3′ |
| J12Mar01 | 5'-cgcgggtctcaagctcaggctgaggtgcagctggtggag-3′ |
| J12Mar02 | 5'-cgcgggtctcactgctgaggagacggtgacctgggt-3′ |
| J13Oct01 | 3'-cgcggaagactaagctcaggctgaggtgcagctggtggag-5' |
| J13Oct02 | 3'- cgcggaagactactgctgaggagacggtgacctgggt -5' |
| D09Oct02pICHR1 | 5'-tttggtttacgccacccctacgg-3' |
| Pre-Immune1_Fwd | 5'-ccatctcatccctgcgtgtctccgactcagctaaggtaacgatctgctgaggagacggtgacc-3' |
| Pre-Immune2_Fwd | 5'-ccatctcatccctgcgtgtctccgactcagaagaggattcgatctgctgaggagacggtgacc-3' |
| Immune1_Fwd | 5'-ccatctcatccctgcgtgtctccgactcagtaaggagaacgatctgctgaggagacggtgacc-3' |
| Immune2_Fwd | 5'-ccatctcatccctgcgtgtctccgactcagtaccaagatcgatctgctgaggagacggtgacc-3' |
| IT_Rev | 5'-cctctctatgggcagtcggtgatcaggagctcaggctgaggtg-3' |
